# Isolation, identification, and pathogenicity of a NADC30-like porcine reproductive and respiratory disorder syndrome virus strain affecting sow production

**DOI:** 10.3389/fvets.2023.1207189

**Published:** 2023-07-07

**Authors:** Hao Chang, Jiaying Zheng, Yingwu Qiu, Chuanxin Chen, Qunhui Li, Qianwen Wu, Limiao Lin, Haishen Zhao, Qingfeng Zhou, Lang Gong, Yankuo Sun, Xiangbin Zhang, Heng Wang

**Affiliations:** ^1^Key Laboratory of Zoonosis Prevention and Control of Guangdong Province, College of Veterinary Medicine, South China Agricultural University, Guangzhou, China; ^2^Maoming Branch, Guangdong Laboratory for Lingnan Modern Agriculture, Maoming, China; ^3^National Engineering Research Center for Breeding Swine Industry, South China Agricultural University, Guangzhou, China; ^4^Wen’s Group Academy, Wen’s Foodstuffs Group Co., Ltd., Xinxing, Guangdong, China

**Keywords:** PRRSV, virus isolation and identification, phylogenetic analyses, recombination analyses, pathogenicity

## Abstract

Since it was first reported in 1987, porcine reproductive and respiratory syndrome virus (PRRSV) has caused several economic crises worldwide. The current prevalence of PRRSV NADC30-like stains causing clinical disease outbreaks in Chain is highly concerning. Immunization against and the prevention of this infection are burdensome for farming organizations as the pathogen frequently mutates and undergoes recombination. Herein, the genetic characterization of a NADC30-like strain (termed BL2019) isolated from a farm in Guangdong Province, China, was analyzed and its pathogenicity for piglets and sows was assessed. Results revealed that BL2019 exhibits a nucleotide homology of 93.7% with NADC30 PRRSV and its NSP2 coding region demonstrates the same 131aa deletion pattern as that of NADC30 and NADC30-like. Furthermore, we identified two recombination breakpoints located nt5804 of the NSP5-coding region and nt6478 of NSP2-coding region, the gene fragment between the two breakpoints showed higher homology to the TJ strain(a representative strain of highly pathogenic PRRSV) compared to the NADC30 strain. In addition, BL2019 infection in piglets caused fever lasting for 1 week, moderate respiratory clinical signs and obvious visual and microscopic lung lesions; infection in gestating sows affected their feed intake and increased body temperature, abortion rates, number of weak fetuses, and other undesirable phenomena. Therefore, we report a NADC30-like PRRSV strain with partial recombination and a representative strain of HP-PRRSV, strain TJ, that can provide early warning and support for PRRS immune prevention and control.

## 1. Introduction

The porcine reproductive and respiratory syndrome (PRRS), is an extremely harmful viral disease that causes tremendous economic losses to the swine industry every year.

The etiological agent is a single-stranded positive-sense RNA virus of ~15.4 kb, belonging to the order *Nido*, family *Arteritis*, and genus *Arteritis* (1). The PRRSV genome contains at least 12 overlapping open reading frames (ORFs) (2, 3). ORF1a and ORF1b encode two replicase polyproteins, pp1a and pp1ab, respectively, which are hydrolyzed by a protease into 16 nonstructural proteins (NSPs): NSP1α, NSP1β, NSP2, NSP2TF, NSP2N, NSP3–6, NSP7α, NSP7β, and NSP8–12; ORF 2–7 encode the structural proteins, including five minor envelope proteins (GP2a, E, GP3, GP4, and ORF5a), two major envelope proteins (GP5 and M), and the nucleocapsid protein (N) (3–5).

PRRSVs are divided into two genotypes: type I (Eurpobartevirus Betaarterivirus suid 1) and type II (Ampobartevirus Betaarterivirus suid 2) (6). The nucleotide homology of the two genotypes differs by ~40%; for instance, the nucleotide homology between the Lelystad virus, a representative strain of the type I genotype, and VR-2332, a representative strain of the type II genotype, is only 60% (7).

PRRSV was first reported in the United States in 1987, eventually becoming an epidemic in Europe (8). The CH-1a strain was first isolated in China in 1996 (9). Particularly since 2006 (10), the highly pathogenic PRRSV JXA1 strain caused massive economic losses to the pig industry, rapidly spreading across the country (11). The PRRSV NADC30 strain was first successfully isolated in the United States in 2008 (12). This strain is a type II PRRSV with clinical manifestations of fever and low mortality. As a member of the Arteritis virus family, PRRSV is characterized by high mutability and recombination rates (4); therefore, it can generate new strains with varying virulence. In 2013, PRRSV strains with a unique genetic background that were frequently identified in south-central China demonstrated the highest nucleotide similarity to NADC30 PRRSVs and were termed NADC30-like PRRSVs. Compared with classical PRRSV and HP-PRRSV, the most striking feature of NADC30-like PRRSVs are the three noncontiguous amino acid deletions at NSP2 (amino acids 322–432, 483, and 504–522), with a total of 131 amino acid deletions (13–15). Although NADC30 is moderately pathogenic, the pathogenicity of different NADC30-like PRRSVs varies significantly. For example, JL580, a representative of NADC30-like PRRSVs, was substantially more pathogenic than the NADC30 and CH-1a strains; the affected pigs showed severe clinical signs with a 100% lethality rate (14). Another NADC30-like PRRSV strain, HNjz15, isolated from the Henan Province in 2015 exhibited the characteristic clinical signs of PRRSV infection, indicating considerably lower pathogenicity than JL580 (16).

The rapid evolution and recombination of PRRSV yielded novel and more virulent viruses, causing clinical PRRS outbreaks (17–19). This study aimed to describe the genetic characteristics of a recombinant type II PRRSV derived from NADC30-like and a TJ strain belonging to HP-PRRSV and to analyze its pathogenicity in piglets and sows.

## 2. Materials and methods

### 2.1. Sample collection and detection

In September 2019, a typical clinical PRRS outbreak occurred at a pig farm in Guangdong, China. The affected nursery pigs exhibited significant respiratory signs, poor growth performance, and sow abortion. Written informed consent was obtained from the owners of the animals for their inclusion in this study. The lung tissue samples of sick pigs in this farm were collected and ground using a lyophilizer (JXFSTPRP-CLN-48, Jingxin, Shanghai, China). Total RNA was extracted from the supernatant using an RNeasy kit (Magen, Shanghai, China) and assayed using real-time polymerase chain reaction (RT-PCR) 2 × AceQ Universal U kit + Probe Master V2 PCR assay kit (Vazyme Biotech, Nanjing, China). PRRSV assays were performed using GP5-specific primers designed and maintained in our laboratory (Table 1).

**Table 1:** The GP5 primer sequence used in the RT-PCR in this study.

Primer	Amplified fragment	Primer sequences
Detection primers	GP5-F	ACCTGAGACCATGAGGTGGGC
	GP5-R	GCCAGAATGTACTTGCGGCCTA

### 2.2. Virus isolation and identification

PRRSV-positive samples were diluted using culture media, filtered through 0.22-μm filters, and inoculated onto primary alveolar macrophages (PAMs) for virus isolation. Cells were cultured in RPMI 1640 media (Fisher Scientific, Waltham, MA, United States) containing 10% fetal bovine serum at 37°C in a 5% humidified CO_2_ atmosphere. PRRSV presence was confirmed by daily cytopathological observation and an indirect immunofluorescence assay (IFA). The supernatant was collected and inoculated into MARC-145; following continuous incubation, the isolated strain was assayed to determine virus titer as well as growth kinetics via RT-PCR and IFA. IFA experiments were performed using a PRRSV N protein mouse monoclonal antibody prepared in our laboratory and an Alexa Fluor 488 goat anti-mouse immunoglobulin G (Abcam, United Kingdom).

### 2.3. Genome sequencing and genetic analysis

The whole genome of PRRSV BL2019 was amplified using specific primers; each PCR product was purified using EasyPure Quick Gel Extraction Kit (Transgen, Beijing, China) and sequenced using Songon (Shanghai, China). Sequences of the untranslated region were sequenced using 3’-RACE Kit and 5’-RACE Kit (Songon, Shanghai, China). All the primers used to amplify PRRSV translated regions sequence were designed and preserved in our laboratory. The BL2019 overlapping fragment sequences were sequenced for assembly using DNASTAR program. Phylogenetic analysis was performed using the MEGA11 phylogenetic neighbor-joining method. The PRRSV genome sequences obtained in this study were deposited in the GenBank database under the accession number OQ735301.

### 2.4. Recombination analysis

Recombinant events were evaluated based on the Recombination Detection Program 4 (RDP4), Chimaera, BootScan, 3Seq, GENECONV, MaxChi, and SiScanto (20). Furthermore, a potential recombination event was considered to have occurred when at least six of the seven detection methods were positive. Similarity comparisons were further performed using SimPlot version 3.5.1 within a 500 bp sliding window along the genomic alignment (20-bp step) (21). The complete BL2019 genome was selected as the query sequence, and the reference sequences were strains JXA1 (EF112445), TJ (EU860248), and CH-1a (AY032626) for China and strain NADC30 (JN654459) for North America.

### 2.5. Animal trials for pathogenicity analyses of the PRRSV BL2019

To assess the pathogenicity of BL2019 PRRSV, we purchased piglets and sows for the experiment from Jada pig farm in Shangbo village, Liantang Town, Gaoyao District, Zhaoqing City, Guangdong Province, China. Their breed, number and grouping in the experiment with the 3R principle. The animal-based experiments performed in this study were approved by the Animal Ethics Committee of South China Agricultural University and conducted under the guidance of the South China Agricultural University Institutional Animal Care and Use Committee (SCAU-AEC-2023C010).The experiment were conducted in appropriate laboratory setting that fulfill the national requirements and comply with the welfare principle of animal. PRRSV antigen- and antibody-negative piglets (*n* = 20; 35 days of age) were randomly divided into two groups: BL2019-inoculated piglets (*n* = 10; each piglet in this group was intramuscularly injected with 2 mL BL2019 virus containing 2 × 10^5^ TCID50) and control group of mock-inoculated piglets (*n* = 10; each piglet in this group was intramuscularly injected with 2 mL Dulbecco’s modified Eagle’s media). PRRSV antigen- and antibody-negative sows (*n* = 6; 85 days of gestation) were randomly divided into two groups: BL2019-inoculated sows (*n* = 3; each sow in this group was intramuscularly injected with 2 mL BL2019 virus containing 2 × 10^5^ TCID50) and control group of mock-inoculated sows (*n* = 3; each sow in this group was intramuscularly injected with 2 mL of Dulbecco’s modified Eagle’s media). Each group was reared independently and provided feed and water *ad libitum*. Following inoculation, the body temperature, body weight, and feed intake of pigs in each group were monitored daily and their clinical signs were recorded. After inoculation, serum was collected from piglets on days 0, 3, 5, 7, and 14 after inoculation and from sows on days 0, 3, 5, 7, 14, 21, 28, and 35 and tested for PRRSV N-specific antibodies using commercial IDEXX HerdChek PRRS X3 enzyme-linked immunosorbent assay kit (Westbrook, ME, United States). The viral copy number was determined using the collected sera RT-PCR to monitor detoxification and detect viremia. In addition, sow farrowing data, including the total litter size, healthy litter size, and abortions, were recorded. Furthermore, umbilical cord tissue was collected from newborn piglets to test the viral load. The piglets were euthanized 14 days after disease onset and then dissected. Pathological changes in the lungs were observed, and lung samples were collected, fixed using 4% paraformaldehyde, and subjected to paraffin-embedded sectioning and hematoxylin and eosin (H&E) staining. The stained sections were subsequently under a light microscope to observe the microscopic histopathology of the lungs. Partial samples of the heart, liver, spleen, lungs, kidneys, submandibular lymph nodes, thymic lymph nodes, and inguinal lymph nodes of the piglets were also collected following autopsy to detect the toxin load of each organ via RT-PCR, as described above.

### 2.6. Statistical analysis

Experimental data were analyzed for significance using GraphPad Prism version 5.0 and are expressed as mean ± standard deviation. *p* < 0.05 was considered significant, and *p* < 0.01 or *p* < 0.001 was considered highly significant.

## 3. Results

### 3.1. Virus isolation and identification

**Figure 1:**
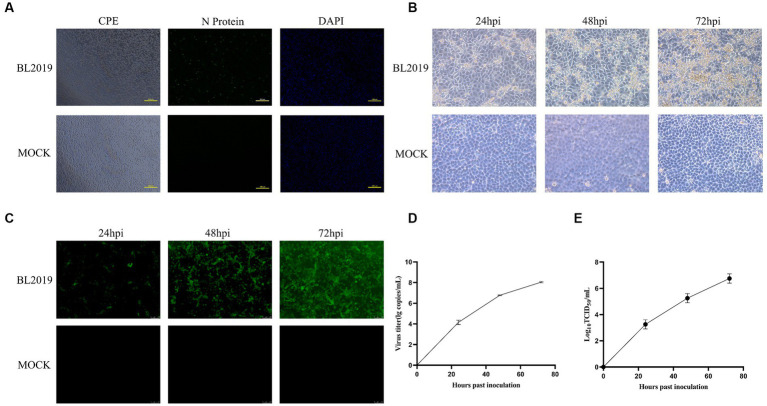
**(A)** Lesions in PAMs after infection and IFA detection with monoclonal antibodies against PRRSV N protein. **(B)** MARC-145 cytopathic lesions at 24, 36, and 72 h postinfection (hpi). Magnification, ×200. **(C)** IFAs showing the reactivity of a monoclonal antibody against PRRSV N protein to BL2019 strain infected at 24, 36, and 72 hpi. **(D)** Viral titer detection of BL2019 strains at 24, 36, and 72 hpi. **(E)** Growth kinetics of the BL2019 strain.

Nucleic acid testing was performed on the lung tissue samples collected from suspected diseased pigs in pig farms. The lung tissue samples positive for PRRSV were processed aseptically and inoculated with PAMs for virus isolation, and the isolated strain was inoculated into MARC-145 cells for biological characterization. PRRSV was successfully isolated from the lung tissue samples (Figure 1A). The biological characterization of the virus revealed more obvious cytopathic lesions with increasing infection time, mainly manifesting as cell aggregation, accumulation, crumpling, rupture without wall apposition, and other lesions, whereas the uninoculated cell group exhibited no cytopathic conditions and the cells grew tightly (Figure 1B). IFA revealed that cells exposed to the virulent strain produced a distinct red fluorescent signal and showed an increased positive fluorescence signals with increasing infection time, whereas the control group exhibited no fluorescence (Figure 1C). In addition, the virus titer and nucleic acid proliferation are shown in Figures 1D,E. Through isolation and identification, we obtained a PRRSV strain named BL2019.

### 3.2. Phylogenetic analysis

**Figure 2:**
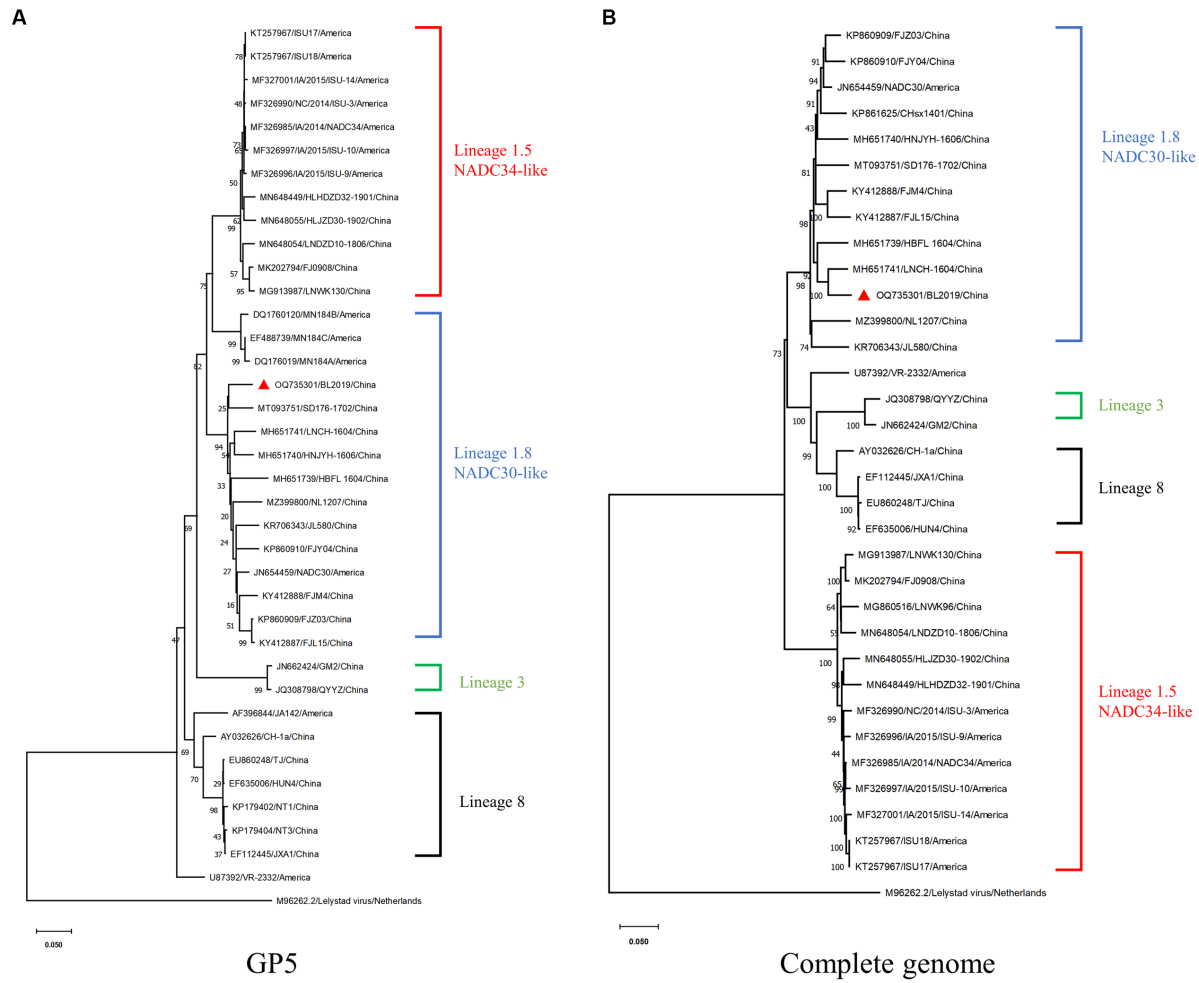
Phylogenetic and recombination analysis of BL2019. **(A)** Phylogenetic trees constructed based on the ORF5 gene of the BL2019 strain with 30 reference PRRSV strains. **(B)** Phylogenetic trees constructed based on the complete gene of the BL2019 strain with 30 reference PRRSV strains. Phylogenetic trees were constructed using the distance-based neighbor-joining method with 1,000 bootstrap replicates in MEGA11.

**Figure 3:**
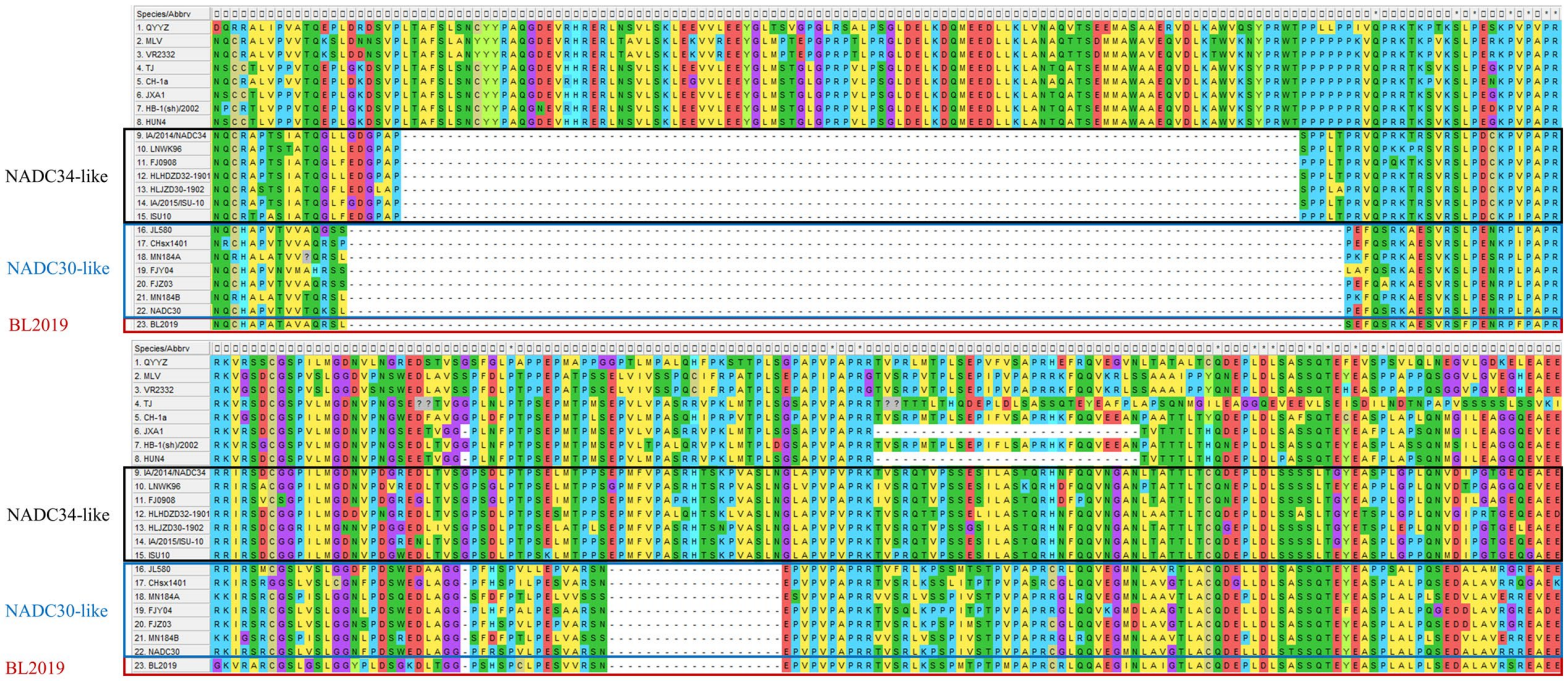
Alignment of the deduced amino acid sequence based on the NSP2 gene. The alignment of the BL2019 strain is marked red.

Whole-genome sequencing revealed that the length of PRRSV BL2019 was 15,022 bp. To further evaluate the evolutionary relationship between the BL2019 isolate and other PRRSVs, the ORF5 and genome-wide genes of the BL2019 isolate and the reference strain were analyzed. BL2019 exhibited 93.7% nucleotide homology with NADC30 and 91.7% nucleotide homology with representative strain of the NADC30-like, strain CHsx1401 (Table 2). The phylogenetic trees based on ORF5 and the whole genome classified the isolate BL2019 as a NADC30-like sublineage (Figures 2A,B). Compared with the reference strain, the NSP2 amino acid sequence of strain BL2019 showed a 131-amino acid (111 + 1 + 19) discontinuous deletion pattern unique to NADC30-like sublineage strains (Figure 3), suggesting that the isolated BL2019 strain belongs to the NADC30-like sublineage.

**Table 2:** Complete genome similarity analysis across different endemic strains in China (%).

Strains	NADC30	VR2332	QYYZ	JXA1	CH-1a	CHsx1401	TJ	BL2019
NADC30	***	86.6	82.9	84.7	85.7	95.7	84.6	93.7
VR2332	15.2	***	86.3	89.6	91.5	85.7	89.7	85.2
QYYZ	20.1	15.6	***	87.9	88.7	82.2	87.9	82.0
JXA1	17.7	11.5	13.6	***	95.2	83.9	99.5	84.1
CH-1a	16.4	9.2	12.5	5.1	***	84.7	95.3	84.5
CHsx1401	4.4	16.4	21.0	18.8	17.7	***	83.8	91.7
TJ	17.8	11.4	13.5	0.6	4.9	18.9	***	84.0
BL2019	6.7	17.0	21.3	18.5	17.9	9.0	18.6	***

### 3.3. Recombination analysis

**Figure 4:**
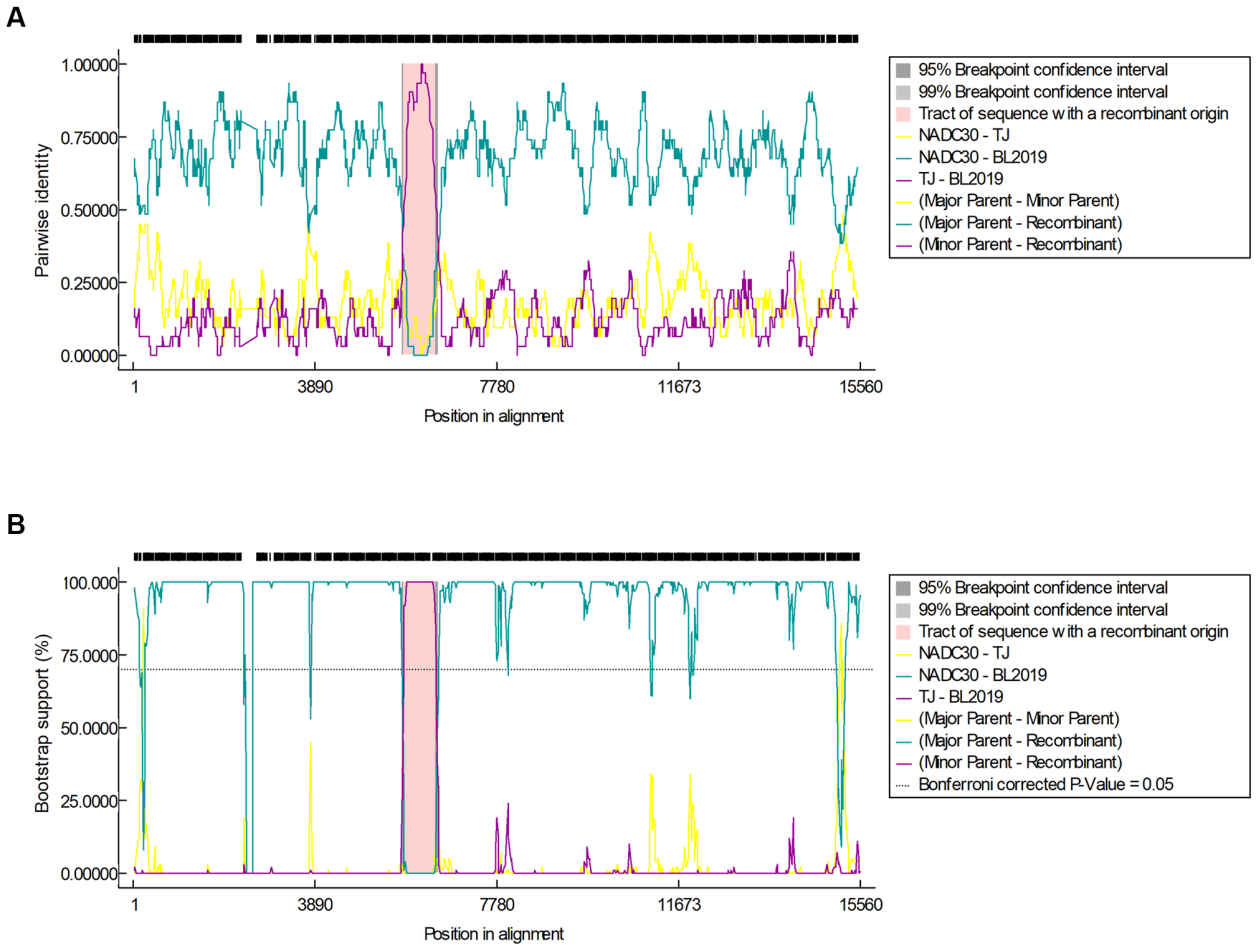
**(A)** Results of the RDP algorithm when the major parent is NADC30 and the minor parent is the TJ strain when analyzed with the RDP4 software. **(B)** Results of the Bootscan algorithm when the major parent is NADC30 and the minor parent is the TJ strain when analyzed with the RDP4 software.

**Figure 5:**
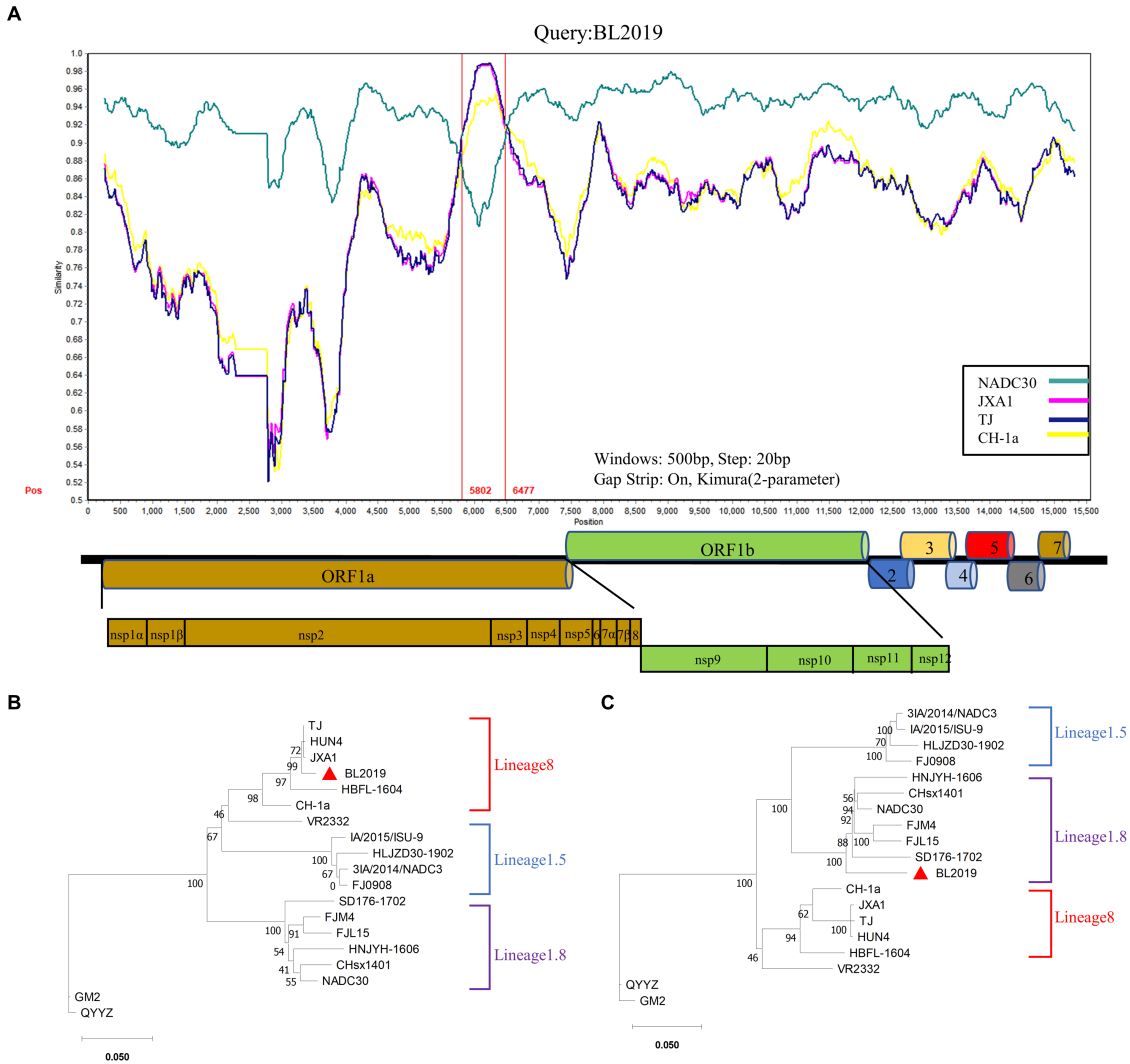
Genomic recombination analysis of BL019 using SimPlot. **(A)** The complete genome of BL2019 was chosen as a query sequence. Solid red lines indicate the recombination breakpoints; the locations are shown at the bottom. The sequence between the solid red lines is the minor parental region, whereas the rest is the major parental region. A complete genome structure is shown under a similarity plot with reference to JXA1, of which the positions of major ORFs are indicated. **(B)** Phylogenetic analysis based on the deduced amino acid sequences of the minor parental region. **(C)** Phylogenetic analysis based on the deduced amino acid sequences of the major parental regions. Phylogenetic trees were constructed using the distance-based neighbor-joining method with 1,000 bootstrap replicates in MEGA11. Numbers along the branches are bootstra*p* values. The scale bar indicates nucleotide substitutions per site.

The recombination analysis was conducted using RDP4 and further confirmed using SimPlot version 3.5.1. The RDP4-based analysis revealed that when NADC30 and the TJ strain were used as the major and minor parents, respectively, a segment of gene exhibiting a high homology with the TJ strain was detected in the BL2019 sequence, and positive results were obtained using all the seven detection methods (Figure 4; Table 3). Two recombination breakpoints were identified within the BL2019 genome at nt5804 of the NSP5-coding region and nt6478 of the NSP5-coding region (Figure 5A). These breakpoints divided the BL2019 genome into three regions, with the middle region demonstrating the highest homology with TJ PRRSV (lineage 8.7), a derivative of the reference strain HP-PRRSV (Figures 5A,B); the rest of the genomic sequence was highly similar to NADC30 PRRSV (lineage 1.8) (Figure 5C).

**Table 3:** Summary of crossover events in the BL2019 strain identified by RDP4.

Recombined virus	Parental virus		Score for the seven detection methods embedded in RDP4						
	Major	Minor	RDP	GENECONV	Boot Scan	Max Chi	Chimaera	Si Scan	3Seq
BL2019	NADC30	TJ	7.354 × 10^−64^	8.858 × 10^−61^	4.758 × 10^−65^	7.068 × 10^−17^	4.113 × 10^−18^	7.409 × 10^−13^	6.661 × 10^−14^

The *p* value cut-off is set at 0.05. *p* < 0.05 indicates the recombination events are significant.

Thus, the PRRSV isolate BL2019 is a recombinant virus resulting from recombination between NADC30 PRRSV and the Chinese HP-PRRSV-derived TJ strain.

### 3.4. Pathogenicity in piglets

**Figure 6:**
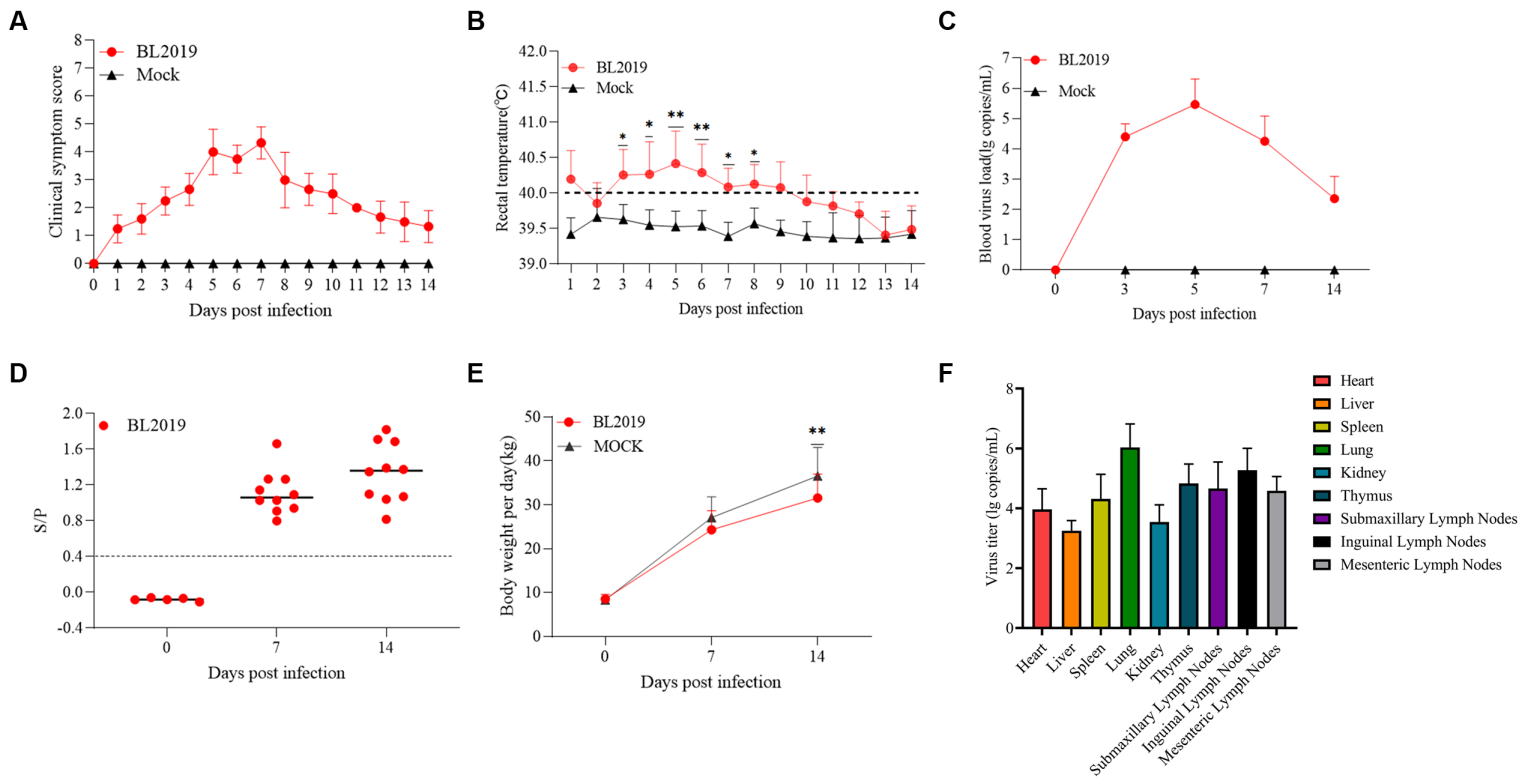
Pathogenicity results of piglets. **(A)** Clinical scores of piglets in each group during the whole experiment. **(B)** Body temperature changes of piglets in each group. **(C)** Body weight change of piglets in each group during the experiment. **(D)** Viral load detection in the blood. **(E)** PRRSV-specific antibody levels were detected in piglets of each group during the challenge study. **(F)** Viral load in the heart, liver, spleen, lungs, kidneys, submandibular lymph nodes, thymic lymph nodes and inguinal lymph nodes of piglets. Significant differences are marked with asterisks: ***p < 0.001; **p < 0.01.

**Figure 7:**
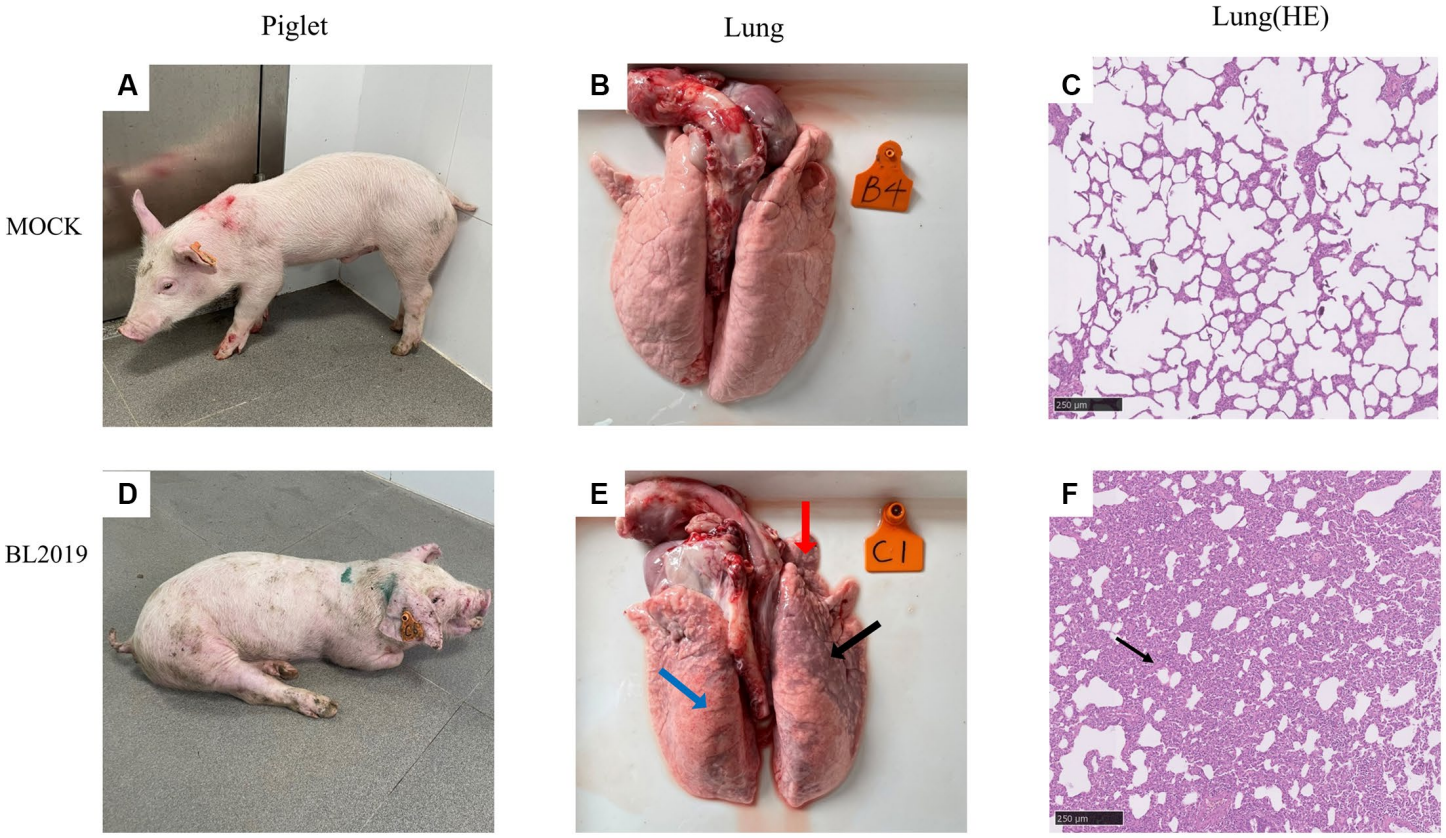
**(A–C)** Clinical pictures of pigs in the control group, lung autopsy pictures, and microscopic pictures of H&E staining of lung tissues. **(D–F)** Clinical pictures of pigs in the BL2019-inoculated group, lung autopsy pictures, and microscopic pictures of H&E staining of lung tissues.

Piglets in the BL2019-inoculated group exhibited obvious fever and other clinical signs, including disheveled coat, difficulty breathing, and cyanotic purple skin (Figures 6A,B, 7D). Viremia monitoring revealed that viremia in piglets peaked at day 5, gradually decreasing thereafter (Figure 6C). Moreover, on days 7 and 14 (Figure 6D), pigs in the BL2019-inoculated group displayed increased antibody levels in the body (Figure 6E). By monitoring the body weight of piglets, it was found that the pigs in the BL2019-inoculated group were observed to gain weight more slowly compared with those in the blank control group.

The piglets were euthanized at the end of the trial and samples of heart, liver, spleen, lungs, kidneys, submandibular lymph nodes, thymic lymph nodes, and inguinal lymph nodes were collected to assess the viral load in these organs. The results revealed that the lungs exhibited the highest viral load, followed by the submandibular lymph nodes and thymus, whereas the lowest viral load was detected in the liver (Figure 6F).

Piglets included in the experiments were euthanized on the 14th day for observation, and the lesioned tissues were H&E stained. Ocular lesion analysis of the lung tissues revealed the presence of normal lung tissue in the control group (Figure 7B) and significant lesions in the lungs of piglets in the BL2019-inoculated group. The red, blue, and black arrows in Figure 7 highlight a fleshy lung lesion, diffuse hemorrhagic spot, and bruised lung lesion, respectively. H&E staining indicated the presence of interstitial pneumonia signs, such as interval thickening and inflammatory infiltration, in the alveoli of the BL2019-inoculated piglets, wherein the alveolar wall was infiltrated by numerous macrophages and few lymphocytes and neutrophils (shown using black arrow) (Figure 7F); however, the alveoli of the control piglets were normal.

### 3.5. Pathogenicity in gestating sows

**Figure 8:**
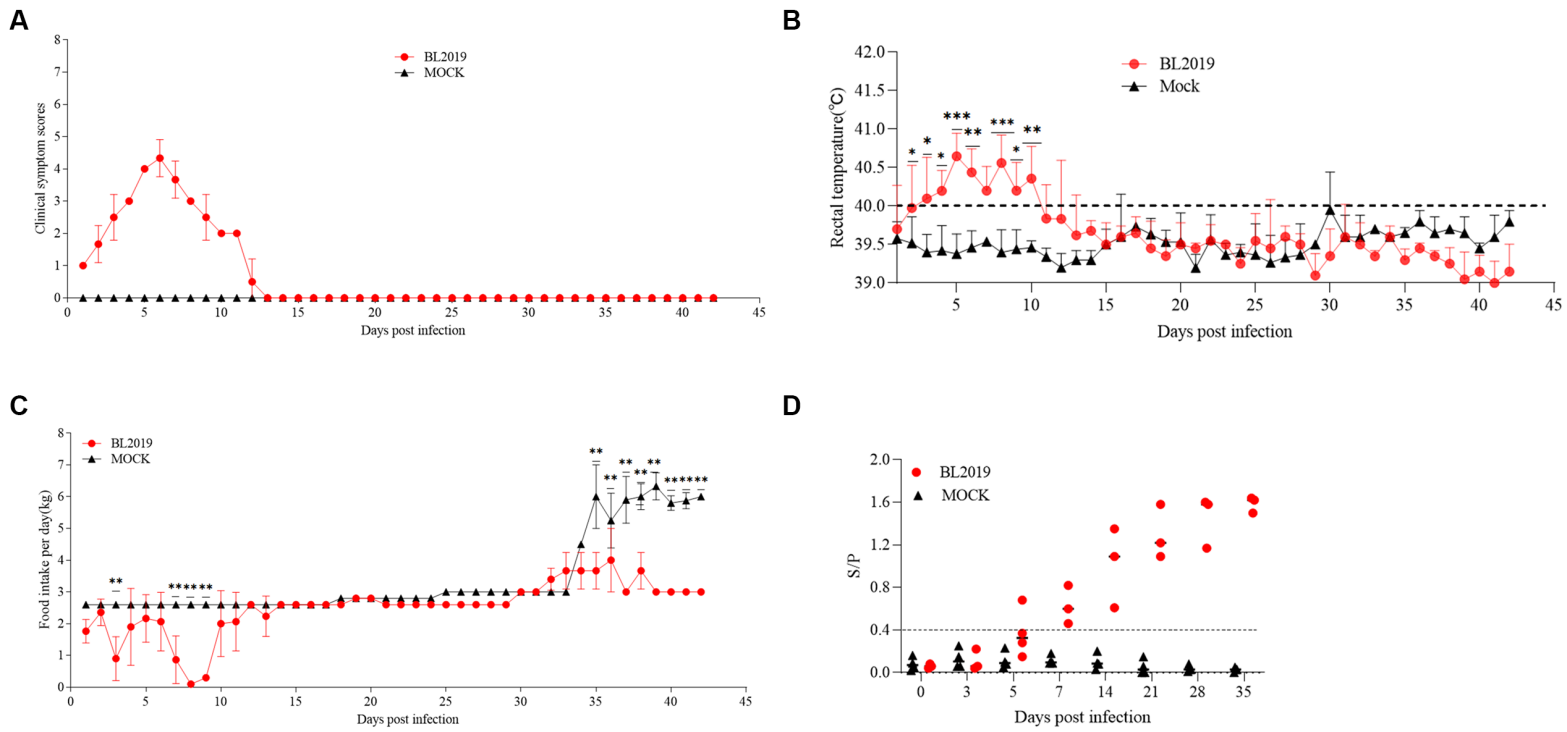
Pathogenicity results in pregnant sows. **(A)** Clinical scores of pregnant sows after a challenge during the entire experiment. **(B)** Body temperature change in pregnant sows in each group after the challenge. **(C)** Food intake changes in pregnant sows in each group after the challenge. **(D)** PRRSV-specific antibody level was detected in each group during the challenge study. Significant differences are marked with asterisks: ***p < 0.001; **p < 0.01.

**Figure 9:**
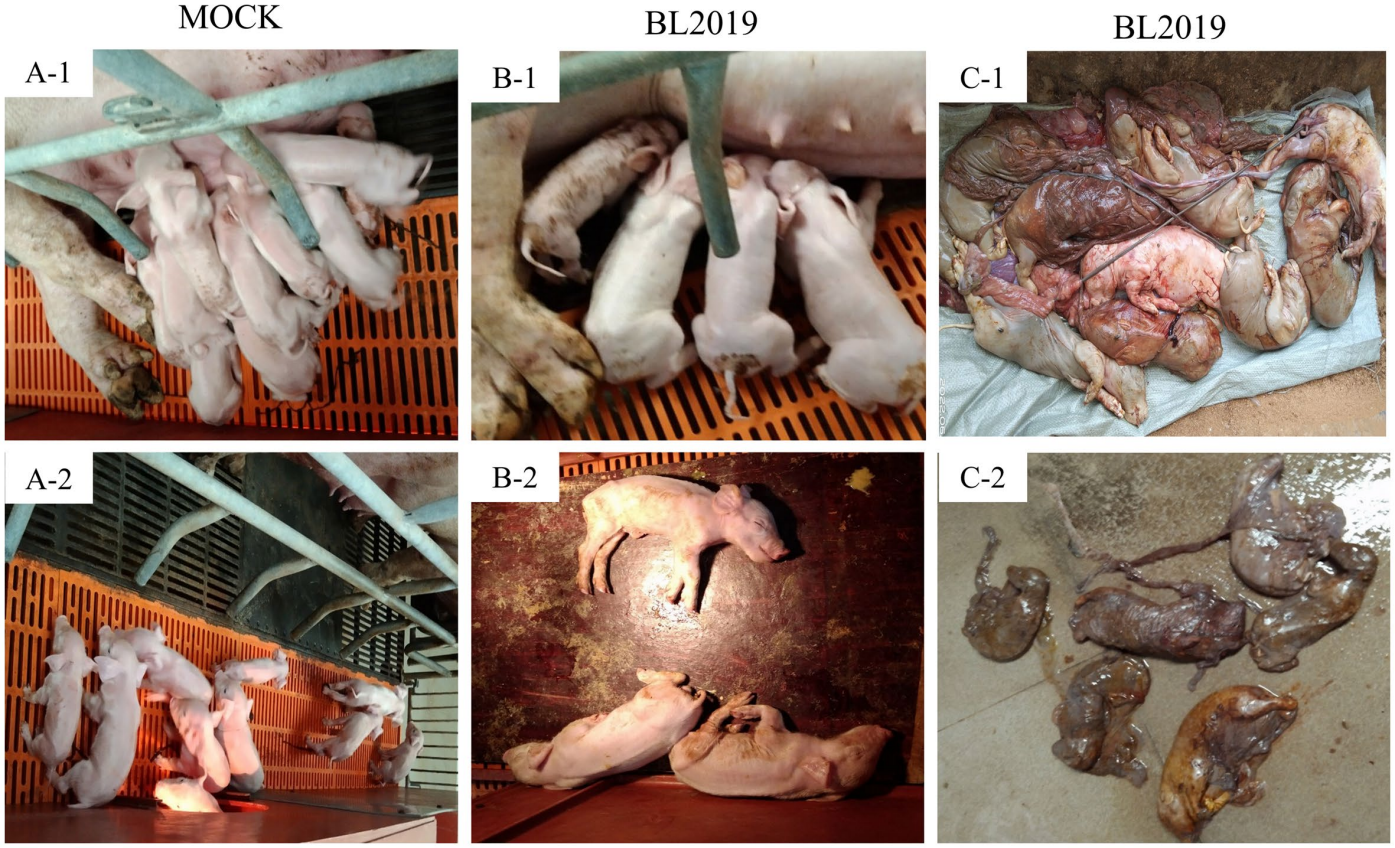
Clinical photographs of sow farrowing. (A-1, A-2) Healthy piglets in control group (B-1, B-2) Surviving piglets in BL2019-inoculated group (C-1) Aborted piglets in BL2019-inoculated group (C-2) Mummified fetuses in BL2019-inoculated group.

The entire trial lasted 42 days. The clinical symptoms of the BL2019-inoculated sows were more serious during days 5–10 until day 12, when the symptoms gradually disappeared (Figure 8A). Body temperature monitoring revealed fever in gestating sows following BL2019 inoculation, which was more obvious at days 4–9 until day 11, when the body temperature gradually returned to normal (Figure 8B). In addition, BL2019-inoculated sows exhibited considerably lower feed intake than control sows in the first 10 days following BL2019 inoculation, and the increase in feed intake was significantly lower in BL2019-inoculated sows than that in control sows from 32 to 33 days after BL2019 inoculation (Figure 8C). PRRSV antibody level analysis revealed the appearance of antibodies in the sows of the BL2019-inoculated group from day 5, with their level gradually increasing from day 7 and reaching a plateau at days 21, 28, and 35 (Figure 8D). Gestational sow farrowing data demonstrated that three sows in the control group had normal farrowing performance, with 11/13/14 piglets, including 11/12/13 healthy piglets, and no sow abortion or stillbirth (Figures 8, 9; Table 4). The cord blood test of the farrowing piglets was negative for PRRSV. Nevertheless, among the three sows in the BL2019-inoculated group, one experienced abortion and the remaining two gave birth to 10/12 piglets, of which only 3/2 were healthy. In addition, the cord blood of the piglets was PRRSV positive (Figure 9; Table 3).

**Table 4:** Farrowing data of sows during the experiment.

Groups	MOCK	BL2019
Challenge virus	DMEM	Virus cultures
*Piglets, number per litter*		
Total born	11/13/14	0/10/12
Live-born	11/12/13	0/3/2
Stillborn	11/12/13	0/3/2
Mummified	0/0/0	0/7/10
Light (<1 kg)	0/1/1	0/3/2
Sow amblosis	0/3 (0%)	1/3 (33.3%)
Sow survival	3/3	3/3
Fetal virus load	None	+

## 4. Discussion

Since 2013, NADC30-like has undergone widespread recombinant mutation, becoming prevalent in China (22) and consequently reducing the cross-protection offered by existing vaccines against this strain. Thus, PRRSV prevention and control in China has become more challenging. Therefore, a large number of clinical samples have been collected over the past few years to investigate PRRSV prevalence. In a clinical sample collected in 2019, we isolated a mutant strain with a recombinant fragment, named BL2019. Following whole-genome sequencing analysis, BL2019 was found to be 93.7% homologous to NADC30, and an evolutionary tree was constructed based on ORF5 and whole-gene sequences. BL2019, along with the recently identified NADC30-like PRRSVs, was classified into lineage 1.5.

NSP2 is one of the most variable regions in the entire PRRSV genome and can serve as a molecular marker. For instance, HP-PRRSV lacks 30 amino acids at NSP2 (1 + 29 amino acids), whereas NADC30 lacks 131 amino acids compared with PRRSV (111 + 1 + 19 amino acids), and the NSP2 protein of NADC34-like strains lacks 100 amino acids at 328–427. After analyzing the NSP2 amino acid sequence of BL2019, we found that the “111 + 1 + 19” amino acids in the NSP2 gene enhanced its resemblance to the NADC30-like strain. In addition, NSP2 inserts and deletions (indels) characterizing 75 discontinuous amino acids (476–552) or 120 continuous amino acids (628–747) deletion in NADC30-like strains were regularly identified (23, 24), indicating a high complexity of the PRRSV that is causing the ongoing epidemic. Considering the high variability of the PRRSV NSP2 gene, these INDELs certainly increase the genetic diversity of PRRSV, thereby continuously threatening the development of the pig industry.

Since its discovery, NADC30-like PRRSV has demonstrated an ability to extensively recombine with multispectral strains. However, the exact mechanism of genetic recombination in PRRSV remains unknown. Nevertheless, it is presumed that the recombination sites in the viral genome are randomly present, as different recombinant strains have been identified with different breakpoints and the frequency of recombination is increasing (25). Although most recombinant NADC30-like PRRSVs exhibit moderate pathogenicity (26), there is a possibility of generating highly pathogenic recombinant strains. In addition, the coexistence of multiple PRRSV on the same farm is a relatively common phenomenon nowadays, which increases the chances of recombination of different PRRSV strains, especially the NADC30-like strain. Further recombination analysis identified two recombination breakpoints located in the nt5804 of the NSP5-coding region and nt6478 of the NSP5-coding region, and they had higher nucleotide homology with the TJ strain, a representative strain of highly pathogenic PRRSV(HP-PRRSV). We further investigated whether the recombination in this gene fragment affected the pathogenicity of the virulent strain. The pathogenicity study of piglets and gestating sows revealed that piglets in the BL2019-inoculated group exhibited clinical signs such as fever, depression, disheveled coat, cyanosis, and purple bodies. Piglets in the BL2019-inoculated group demonstrated reduced feed intake and slower growth than those in the control group; however, the experimental piglets survived the entire test cycle. Following the autopsy, there were obvious signs of lung hemorrhage, and the H&E staining of the lung pathology sections revealed that the BL2019 infection damaged the alveoli, resulting in inflammatory infiltration and parenchymal lesions. Gestating sows in the BL2019-inoculated group showed abortion and stillbirth. Accordingly, BL2019 PRRSV affected piglet growth and the reproductive performance of sow performance but was significantly less pathogenic than the TJ strain (27). We speculate that this phenomenon occurred because of the genetic recombination ability of this strain. NSP4 has been proven to be the major protease of PRRSV (i.e., a serine protease similar to 3C) that is involved in the processing of all NSPs except NSP1a/NSP1p, NSP1p/2, and NSP2/3 (25, 28). In future studies, we aimed to determine how recombination in this gene fragment affects the pathogenicity of the strain. Additionally, owing to the coexistence of multiple PRRSVs, there is an urgent need to monitor the prevalence of NADC30-like PRRSV, paying close attention to their recombination potential; gain insights into the pathogenicity of different recombinant strains; and design effective PRRSV prevention and control programs.

In conclusion, we isolated and reported a new strain of NADC30-like PRRSV, named BL2019. Although no piglets died during the pathogenicity tests, autopsy observations revealed that BL2019 caused lung lesions in piglets. In addition, this strain can cause abortion and weak fetus in gestating sows as well as other phenomena that affect the reproductive performance of sows. Therefore, if a large-scale epidemic of this strain occurs, it may pose a new threat and challenge to pig breeding.

## Data availability statement

The datasets presented in this study can be found in online repositories. The names of the repository/repositories and accession number(s) can be found at: https://www.ncbi.nlm.nih.gov/ OQ735301.

## Ethics statement

Written informed consent was obtained from the host to facilitate our collection of clinical samples in this study. Our animal experiments were approved by the Animal Ethics Committee of South China Agricultural University and conducted under the guidance of the South China Agricultural University Institutional Animal Care and Use Committee (SCAU-AEC-2023C010).

## Author contributions

HW and XZ conceived and designed the study and reviewed the manuscript and edited it. HC, JZ, and YQ devised the experimental methods. HC and JZ curated the data. HC, YQ, QL, QW, LL, HZ, QZ, LG, and YS provided resources and performed the experiments. HC prepared the original manuscript draft. All authors have read and approved the final manuscript.

## Funding

This research was funded by the National Natural Science Foundation of China (32102704 and 31872489), Start-up Research Project of Maoming Laboratory (2021TDQD002), and China Agriculture Research System of MOF and MARA (cars-35).

## Acknowledgments

The authors thank Wen’s Group Academy and Wen’s Foodstuffs Group.

## Conflict of interest

YQ, QL, LL, HZ, and QZ were employed by Wen’s Foodstuffs Group Co., Ltd.

The remaining authors declare that the research was conducted in the absence of any commercial or financial relationships that could be construed as a potential conflict of interest.

## Publisher’s note

All claims expressed in this article are solely those of the authors and do not necessarily represent those of their affiliated organizations, or those of the publisher, the editors and the reviewers. Any product that may be evaluated in this article, or claim that may be made by its manufacturer, is not guaranteed or endorsed by the publisher.
