# Supplementary material for: Isolation, identification, and pathogenicity of a NADC30-like porcine reproductive and respiratory disorder syndrome virus strain affecting sow production
Source: Front Vet Sci. 2023 Jul 7;10:1207189. doi: 10.3389/fvets.2023.1207189 (PMC10360194; doi:10.3389/fvets.2023.1207189)
Supplement: Supplementary file 1 [file Table_1.DOCX]

Supplementary Material

**Isolation, identification and pathogenicity of a NADC30-like porcine reproductive and respiratory disorder syndrome virus strain affecting sow production**

**Hao Chang^1,3,4*^, Jiaying Zheng^1*^, Yingwu Qiu^1,2^, Chuanxin Chen^1^, Qunhui Li^2^, Qianwen Wu^1^, Limiao Lin^2^, Haishen Zhao^2^, Qingfeng Zhou^2^, Lang Gong^1,3^, Yankuo Sun^1,3,4^, Xiangbin Zhang^1,2*^, Heng Wang^1,3,4*^**

*** Correspondence:** Heng Wang: [wangheng2009@scau.edu.cn](mailto:wangheng2009@scau.edu.cn)

Xiangbin Zhang: zhangxb@scau.edu.cn

# Supplementary Tables

Table 1：Primer sequences used in this study.

| Primer | Amplified ragment | primer sequences |
| --- | --- | --- |
| Whole genome sequencing primers | 1 | 5’-ATGACGTATAGGTGTTGGCTCTA-3’ |
|  |  | 5’-TCGTGTGCAGTAGACCTGGC-3’ |
|  | 2 | 5’-CAGGAGCTGTGACCATTGGC-3’ |
|  |  | 5’-CACCGTACCACTTRTGACTGC-3 |
|  | 3 | 5’-AGGGTTGAGYCYAAYACGTC-3’ |
|  |  | 5’-CTGAGYGACCACAGTYCTAG-3’ |
|  | 4 | 5’-CGRAGYCCCATTGAGCAACC-3’ |
|  |  | 5’-TTCRACGCCAGAGGATGTYAG-3 |
|  | 5 | 5’-GATTGCCCGAATTGGCAAGG-3’ |
|  |  | 5’-TGCCACGGTATCRGCAAAAG-3’ |
|  | 6 | 5’-GAGGAATGCAGCGGGYCAAT-3’ |
|  |  | 5’-TCGCCGTCGACGTTCATCAT-3’ |
|  | 7 | 5’-TCCCCGYCAACCCYGAGAAT-3’ |
|  |  | 5’-TAGGCCTGACTGCCYTAAACAG-3’ |
|  | 8 | 5’-TAGGCCTGACTGCCYTAAACAG-3’ |
|  |  | 5’-CCCGCACAYTCTSGACTTCT-3’ |
|  | 9 | 5’-CTCATGGACAGCTGTGCTTG-3’ |
|  |  | 5’-GCCTGTGTGGGTCATACAC-3’ |
|  | 10 | 5’-GGRCAGAATATCTGTGATGCCA-3’ |
|  |  | 5’-GACYACCGCAACTGATTCCTT-3’ |
|  | 11 | 5’-AGTCCCTCCCACAYGCYTTCAT-3’ |
|  |  | 5’-GCCTCGCTCACCACCTGTTTC-3’ |
|  | 12 | 5’-TGCGCGCYCTRCCRTTCAC-3’ |
|  |  | 5’-CCGAAAGACTCGAACTGAAAC-3’ |
|  | 13 | 5’-GYCCYTTCTTTTCCTCBTGGTT-3’ |
|  |  | 5’-CGGAACCATCAAGCACAACT-3’ |
|  | 14 | 5’-CTATCGTTGGCGGTCGCCYGTCA-3’ |
|  |  | 5’-AATTACGGCCGCATGGTTCTCGC-3’ |

Table 2： PRRSV strains used in this study.

| Strain name | Registration Number | Strain name | Registration Number | Kt257967 | Registration Number |
| --- | --- | --- | --- | --- | --- |
| ISU17 | KT297967 | MN184B | DQ176020 | FJZ03 | KP860909 |
| ISU18 | KT257968 | MN184A | DQ176019 | CHsx1401 | KP861625 |
| ISU-14 | MF327001 | MN184C | EF488739 | FJL15 | KY412887 |
| ISU-3 | MF326900 | SD176-1702 | MT093751 | GM2 | JN662424 |
| NADC34 | MF326985 | LNCH-1604 | MH651741 | QYYZ | JQ308798 |
| ISU-10 | MF326997 | HNTYH-1606 | MH651740 | JA-142 | AF396844 |
| ISU-9 | MF326996 | HBFL-1604 | MH651739 | CH-1a | AY032626 |
| HLHDZD32-1901 | MN648449 | NL1207 | MZ399800 | TJ | EU860248 |
| HLHDZD30-1902 | MN648055 | JL580 | KR706343 | HUN4 | EF635006 |
| LNDZD10-1806 | MN648054 | FJY04 | KP860910 | NT1 | KP179402 |
| FJ0908 | MK202794 | NADC30 | JN654459 | NT3 | KP179404 |
| LNWK130 | MG913987 | FJM4 | KY412888 | JXA1 | EF112445 |
| VR2332 | U87392 | Lelystad virus | M96262 |  |  |
